# *Porcine Rotavirus*: pathogenic mechanisms, host immune responses, and progress toward control

**DOI:** 10.3389/fmicb.2026.1859923

**Published:** 2026-08-13

**Authors:** Jiyu Liu, Yuexin Dong, Zhongxing Xu, Jinyong Zhang, Lianmao Duan, Guangshan Zhu, Nini Ma, Shaonan Zhang, Cong Wang, Xuexia Wen

**Affiliations:** 1 College of Veterinary Medicine, Hebei Agricultural University, Baoding, China; 2 China Animal Husbandry Industry Co., Ltd., Beijing, China; 3 Academy of Military Medical Sciences, Beijing, China; 4 Shandong New Hope Liuhe Group Co., Ltd./Shandong Engineering Research Center of Pig and Poultry Health Breeding and Important Infectious Disease Purification, Qingdao, Shandong, China; 5 Riverstone Farm (Shandong) Co., Ltd., Jinan, China; 6 Key Laboratory of Livestock Infectious Diseases, Ministry of Education, and Key Laboratory of Ruminant Infectious Disease Prevention and Control(East), Ministry of Agriculture and Rural Affairs, College of Animal Science and Veterinary Medicine, Shenyang Agricultural University, Shenyang, China

**Keywords:** epidemiology, immunologic mechanism, pathogenic mechanism, *Porcine Rotavirus*, vaccine development

## Abstract

*Porcine Rotavirus* (*PoRV*), a major pathogen causing diarrhea in piglets and significant economic losses, exhibits high genotypic diversity and poses a risk of cross-species transmission. *PoRV* infection is initiated by the interaction of its outer capsid proteins, VP4 and VP7, with multiple host cell receptors (e.g., sialic acids, integrins), triggering complex processes of endocytosis and membrane penetration that ultimately lead to enterocyte dysfunction and diarrhea. Diverse immune evasion strategies have been evolved by *PoRV*, among which the host interferon responses and cellular protein synthesis are both suppressed by nonstructural proteins including NSP1 and NSP3. Host defense relies on innate immunity (e.g., RIG-I/MDA-5 and TLR3 signaling pathways) and adaptive immunity centered on virus-specific T cells. Epidemiological surveillance indicates the dominance of RVA genotypes in swine populations worldwide. Furthermore, genetic reassortment events between porcine and human strains underscore a significant zoonotic transmission risk, which poses a significant challenge to public health and compromises the efficacy of existing vaccines. Current control measures rely on biosecurity management and vaccination to enhance maternal antibody transfer. However, given the high genetic variability of the virus, the development of novel broad-spectrum vaccine platforms, such as those based on virus-like particles (VLPs) or multi-epitope antigens, is urgently needed. In this review, current research progress is systematically integrated to advance the understanding of *PoRV*-host interactions and provide evidence-based guidance for the development of effective control strategies.

## 1 Introduction

*Rotavirus (RV)*, a dsRNA virus, belongs to the genus *RV* within the family *Reoviridae*. The *RV* genome consists of 11 dsRNA segments, which encode six structural (VP1-VP4 and VP6-VP7) and five nonstructural proteins (NSP1-NSP5) (Flewett et al., 1974). This genome is packaged within a triple-layered particle (TLP). Mature *RV* particles exhibit a TLP structure composed of four structural proteins: VP2, VP6, VP7, and VP4. The VP1, VP2, and VP3 proteins form the inner capsid of the virus particles, and the middle capsid consists of the VP6 protein, which determines the specificity of the serogroup and is the most important immunogenic protein. The outer capsid contains two components: the glycoprotein VP7 and the spike protein VP4. Both VP4 and VP7 mediate the attachment of *PoRV* to host cell receptors and subsequent viral entry. Trypsin-mediated cleavage of VP4 yields VP5* and VP8* fragments, which are critical for membrane penetration and infectivity enhancement. TLP, upon exposure to simulated cytoplasmic levels of low calcium ion concentrations, is converted to transcriptionally active noninfectious double-layer particles (DLP). The VP6-encapsulated DLP contains a transcriptionally active dsRNA-dependent RNA polymerase complex, which transcribes and releases nascent viral mRNA transcripts into the cytoplasm through pores in the DLP capsid (Crawford et al., 2001; Matthijnssens et al., 2012).

Currently, *RV* genotypes are classified into 10 species (designated RVA-RVI) based on comparative sequence analysis of VP6 nucleotide data (Matthijnssens et al., 2012). *RV* strains are further characterized by their G (glycoprotein VP7) and P (protease-sensitive VP4) genotypes.

## 2 Epidemiology

RV displays substantial genetic diversity within swine populations. Since RVA was first identified in 1975, multiple genotypes, including RVB, RVC, RVE, and RVH, have been successively discovered (Matthijnssens et al., 2012). Among these, RVA remains the most prevalent and pathogenic species in pigs. However, recent epidemiological surveys indicate a steady increase in RVC detection rates in diarrheic neonatal piglets (Amimo et al., 2013; Martella et al., 2007a, 2007b). RVB primarily infects adult pigs, and the pathogenic significance of other serotypes remains to be determined (Alekseev et al., 2018; Basera et al., 2010; Chasey et al., 1986; Homwong et al., 2016; Theil et al., 1985). *PoRV* infections exhibit age-dependent patterns. The most severe clinical manifestations occur in neonatal piglets (<2 months old), with mortality rates reaching 95% in unprotected populations. As they age, these infections often transition to subclinical forms. Notably, *PoRV* possesses the zoonotic potential, posing a threat to public health (Alam et al., 2013; Giri et al., 2019; Kaminjolo and Adesiyun, 1994; Legrottaglie et al., 1993).

In recent years, the global epidemic pattern of *PoRV* has undergone significant changes. For porcine RVA, the historically dominant G5P[7] genotype in the Americas has been progressively supplanted by globally prevalent genotypes such as G9P[13] and G9P[23]. Meanwhile, infection rates and genetic diversity of porcine RVC (predominantly G6) and RVB remain underestimated, with detection rates in some regions even surpassing those of RVA (Martella et al., 2007a, 2007b; Miyabe et al., 2020; Niira et al., 2016; Parra et al., 2008; Suzuki et al., 2015; Vlasova et al., 2017). According to the global data compiled by Vlasova et al., 12 G genotypes (G1, G2, G3, G4, G5, G6, G8, G9, G10, G11, G12, G26) and 16 P genotypes (P[1], P[2], P[3], P[4], P[5], P[6], P[7], P[8], P[11], P[13], P[19], P[23], P[26], P[27], P[32], P[34]) have been identified in swine herds, with the distribution of predominant genotypes varying considerably across regions (Vlasova et al., 2017) (Table 1).

**Table 1:** Global distribution of predominant G and P genotypes of *PoRV* in swine herds.

Region	G genotype	P genotype	Genotype combination
North America	G9, G4, G11	P[13], P[7]	G9P[13] (60.9%), G9P[7] (8.7%)
South America (Brazil and Argentina)	G3, G5, G9	P[6], P[13], P[23]	G5P[13], G9P[23]
Africa	G5, G23	P[6], P[8], P[13]	
Europe	G2, G3, G4, G5, G9, G11	P[6], P[7], P[13], P[23]	G3P[6], G4P[6], G5P[6], G4P[7], G5P[7], G9P[7], G9P[13], G9P[23]
Asia	G2, G3, G4, G5, G9, G11	P[6], P[7], P[13], P[23], P[19]	G9P[23], G9P[7]

This dynamic evolution of *PoRV* is driven by multiple factors. First, the widespread use of live attenuated vaccines based on G5P[7] has strongly suppressed homotypic strains through herd immunity, this selective pressure has simultaneously created ecological niches for heterotypic strains such as G9. Notably, genetic recombination between vaccine and wild-type strains continues to generate novel reassortants capable of evading existing immunity; second, cross-species spillover and re-introduction among humans, swine, and cattle further introduce foreign gene segments, increasing genetic diversity. Furthermore, the upgrading of molecular diagnostic techniques from traditional serology to sequencing analysis (Homwong et al., 2016; Marthaler et al., 2013) has allowed the previously underestimated diversity of RVB and high prevalence of RVC to be truly revealed (Marthaler et al., 2013).

Beyond these virological and technological factors, the farming system model also influences the epidemiological characteristics of *PoRV*. Genotype distributions and transmission dynamics differ markedly between intensive and smallholder farming systems. In intensive systems, high-density rearing and batch production lead to high viral transmission efficiency (Boene et al., 2021), and predominant genotypes are highly homologous within the same farm, with transmission relying more on international trade and introduction of breeding animals. In contrast, in smallholder systems, closer contact between swine and humans results in more frequent detection of genotypes highly similar to human strains, such as P[8] (Amimo et al., 2015), and provides more opportunities for cross-species transmission and genetic reassortment. For example, G4P[6] and G9P[13] strains detected in smallholder swine herds in Zambia showed high sequence similarity to both human and porcine strains (Ndebe et al., 2023). These observations indicate that intensive and smallholder farming systems play distinct roles in *PoRV* epidemiology, which, respectively, present distinct challenges: rapid internal transmission in intensive systems and external cross-species introduction in smallholder systems. Consequently, tailoring surveillance protocols and intervention strategies to specific production systems is a critical step toward improving *PoRV* control.

Given the remarkable genetic diversity and continuously evolving epidemiological landscape of *PoRV*, establishing routine genotypic surveillance is imperative. Systematic molecular monitoring will facilitate timely detection of emerging strains and inform vaccine strain updates, providing an essential foundation for effective control strategies (Kumar et al., 2022; Li et al., 2022; Vlasova et al., 2017; Wang et al., 2019).

## 3 Pathogenic mechanism

### 3.1 Interactions between *RV* and host cell receptors

RV pathogenesis begins with viral adhesion and entry, a process driven by sequential interactions between the surface proteins VP4/VP7 and host receptors (Arias et al., 2002; Coulson et al., 1997; Graham et al., 2003; Guerrero et al., 2002; Guerrero et al., 2000; Mendez et al., 1999; Zarate et al., 2000a, 2000b). Key identified cellular receptors include:

- Sialic acid (SA, N-acetylneuraminic acid);
- Histo-blood group antigens (HBGAs);
- Integrins (α2β1, α4β1, αXβ2);
- Heat shock cognate protein 70 (Hsc70).

Sialic acid (SA) is a key attachment factor for a few animal *RVs*, such as porcine OSU and simian RRV (Guo et al., 2021), binding to an open shallow groove (residues 93–208) composed of conserved residues within the VP8 domain of VP4 protein. Notably, some animal and human-derived *RVs* (e.g., Wa and DS1) are SA-independent (Ciarlet et al., 2002a, 2002b; Kuhlenschmidt et al., 1999). Studies have confirmed that HBGAs are key receptors for *RV*: *PoRV* G5P[7] OSU and G9P[13] bind H-type HBGA via VP8, while G4P[6] (Gottfried) binds H/A HBGA. Furthermore, human HBGAs share homology with porcine (primarily types A and H1). The similarity between human and porcine HBGAs may underlie the mechanisms of RV zoonosis and interspecies transmission (Guo et al., 2021).

Several integrins serve as *RV* receptors (Ludert et al., 1998). VP4 has integrin-binding motifs for α2β1 and α4β1, while VP7 binds αxβ2 and α4β1 (Coulson et al., 1997). Some *RV* use α2β1 for attachment; others require α2β1 even after SA binding (Ciarlet et al., 2002a, 2002b; Graham et al., 2003), indicating its role in multiple infection stages. The DGE motif (aa 308–310) in VP5 mediates α2β1 binding (Zarate et al., 2000a, 2000b). Recombinant VP5 directly binds integrin α2. Integrin usage depends on VP4 but not VP7 serotype or NA sensitivity (Graham et al., 2003).

Following initial binding to SA and integrin α2β1, *RV* engages three additional surface proteins: hsc70, integrin αvβ3, and integrin αxβ2 (Graham et al., 2003; Guerrero et al., 2000). The hsc70 interaction is mediated by a VP5 domain (residues 642–659). This interaction, however, demonstrates limited sequence specificity (Jolly et al., 2001). Integrin αvβ3 mediates post-attachment entry of select *RV* strains. This interaction occurs via a conserved CNP motif in VP7 (residues 161–169) (Graham et al., 2003). These steps are essential for the completion of viral entry and have also been confirmed to be important targets for neutralizing antibodies (Ludert et al., 2002).

### 3.2 Cell entry and replication strategies of *RV*

**Figure 1:**
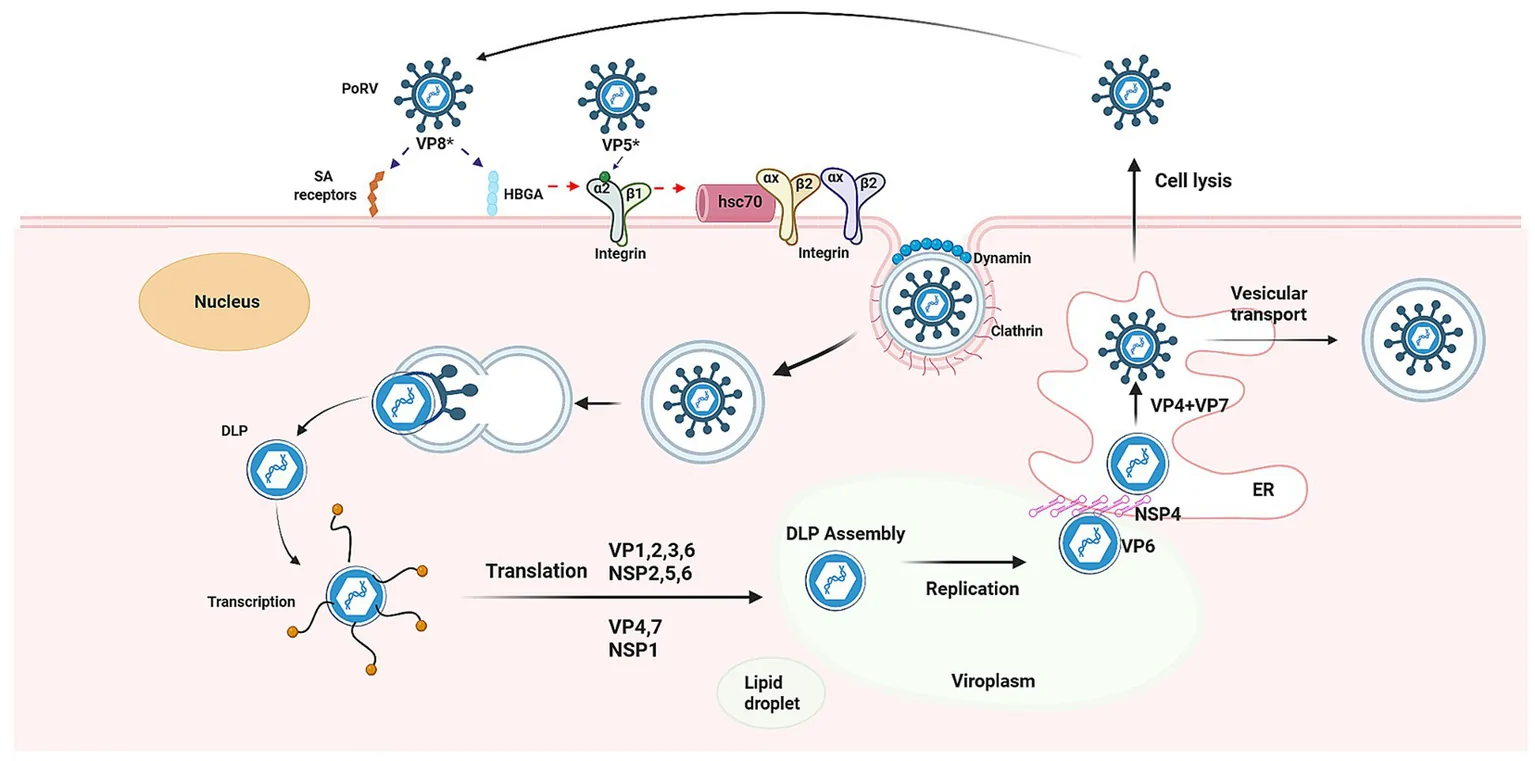
*Porcine Rotavirus* (*PoRV*) invasion and replication: *PoRV* attaches to sialic acid (SA) or histo-blood group antigens (HBGAs) on the host cell surface, interacts with integrins and Hsc70 receptors, and mediates the endocytosis pathway via dynamin and cholesterol-dependent lattice proteins. DLP are released into the cytoplasm, where they undergo mRNA transcription, DLP bind to NSP4 and bud out into the ER, where transient membranes are removed and the TLP mature as the VP4 and VP7 proteins are assembled. The progeny virions are released from the cell via cell lysis or a Golgi-independent non-classical vesicular transport mechanism in polarized epithelial cells.

The consensus mechanism of *RV* cellular entry remains incompletely understood. Strain-dependent entry mechanisms primarily include (1) dynamin and cholesterol-dependent endocytosis, (2) clathrin-mediated endocytosis, and (3) direct membrane penetration (Crawford et al., 2017; Diaz-Salinas et al., 2013; Yamauchi and Helenius, 2013). Electron microscopy evidence and rapid membrane penetration phenomena support the existence of direct entry mechanisms. Intestinal infection initiates when VP8 binds glycolipid receptors (e.g., SA, HBGA) on enterocytes or secretory cells, triggering cellular polarization (Figure 1). Proteolytic cleavage (by trypsin/chymotrypsin) induces VP8 dissociation, exposing VP5 hydrophobic domains—a critical conformational change initiating viral entry. Subsequently, with the assistance of clathrin, VP5 mediates the clustering of receptors (such as Hsc70 and integrins) and the invagination of the cell membrane (Nava et al., 2004). Gangliosides (sialylated sphingolipids) play a critical role in *RV* cellular entry. Functional studies demonstrate that while ganglioside depletion does not affect viral binding, it significantly reduces infectivity without impacting post-entry replication. The *RV*-ganglioside interaction further drives membrane invagination, facilitating the formation of tightly packed vesicles (Martinez et al., 2013).

Upon internalization via endocytic vesicles and transport to the cytoplasm, the viral protein VP5 mediates three critical events: (1) induces heterogeneous vesicle component distribution, (2) enhances membrane permeability, facilitating Ca2 + efflux, and (3) triggers VP7 outer capsid disassembly through calcium depletion (Periz et al., 2013). Acidic pH induces VP5 conformational change—a 180° rotation of its hydrophobic domain forms membrane-permeabilizing trimers that disrupt vesicle integrity. Notably, VP5 alone exhibits membrane poration activity, sufficient for DLP release (Figure 1) (Hyser et al., 2010).

Thereafter, the virus forms viroplasms (containing viral and cellular proteins as well as lipids) in the cytoplasm, which are responsible for: (1) viral mRNA translation, (2) genomic RNA replication, and (3) progeny DLP assembly (Cheung et al., 2010) (Figure 1). NSP4 serves as an intracellular receptor, mediating VP6-coated DLP budding into the ER through transient lipid acquisition. ER-localized maturation involves: (1) envelope loss, (2) VP7 outer layer formation, (3) VP4 spike insertion/activation, culminating in non-lytic (vesicular) or lytic release (Hyser et al., 2010; Pham et al., 2017) (Figure 1). In addition to mediating budding, NSP4 orchestrates calcium signaling. It depletes ER Ca^2+^ stores, thereby activating store-operated calcium entry (SOCE), and also induces the opening of plasma membrane Ca^2+^ channels. Concurrently, NSP4 forms plasma membrane pores, enhancing cation flux, creating a calcium-rich cytoplasmic milieu essential for replication (Crawford et al., 2017).

### 3.3 Intestinal pathologic injury and host compensatory response

*RV* infection is primarily confined to the intestinal mucosa, and the diarrhea it induces results from the combined effects of multiple factors. Traditional mechanisms include (Stanifer et al., 2019): Viral replication directly leads to necrosis of intestinal epithelial cells and loss of associated digestive enzymes, causing malabsorptive diarrhea (Lazear et al., 2019). Simultaneously, the viral enterotoxin NSP4 activates cellular Ca^2+^ signaling, thereby inducing chloride secretion on one hand while promoting serotonin release and stimulating enteric nerves on the other (Hyser et al., 2010). This synergistic action leads to both secretory diarrhea and enhanced intestinal motility (Crawford et al., 2017). While the host responds to the injury through compensatory repair mechanisms such as significantly enhancing protein synthesis in the intestinal epithelium (Rhoads et al., 2007) and increasing crypt cell proliferation (Boshuizen et al., 2005; Davidson et al., 1977). Notably, recent studies suggest that diarrhea itself may represent an active defense strategy of the host. Innate immune signals, such as IFN-*λ*, can actively increase intestinal fluid secretion by regulating ion transport, potentially accelerating viral clearance. This provides a new perspective for understanding the pathophysiology of diarrhea (Hou et al., 2025).

## 4 Host immune response mechanisms

*PoRV* infection triggers a complex host immune response involving coordinated actions of innate and adaptive immunity. Direct immunological data specific to *PoRV*, particularly at cellular and molecular levels, remain relatively limited. Current understanding of RV immune mechanisms has been substantially advanced by studies using gnotobiotic piglets infected with porcine or human RV strains. This model is widely recognized as a powerful tool for investigating RV pathogenesis and immune responses (Saif et al., 1997; Yuan and Saif, 2002; Yuan et al., 2008). Data derived from this model provide essential inference bases for understanding immune protection mechanisms under natural porcine infection conditions. This chapter integrates available evidence with particular attention to its applicability to *PoRV*.

### 4.1 Innate immune response

The innate immune system constitutes the first line of defense against *PoRV* invasion. It is responsible for pathogen recognition, viral clearance, antigen presentation, and initiation of adaptive immunity. Studies using *in vitro* and animal models demonstrate that intestinal barrier disruption, such as EGTA induced tight junction damage, significantly increases *PoRV* susceptibility (Cui et al., 2019), consistent with observations in murine and human cell lines (Hagbom et al., 2020; Moncada et al., 2003). These findings confirm the critical role of epithelial integrity in resistance to infection (Cui et al., 2019).

**Figure 2:**
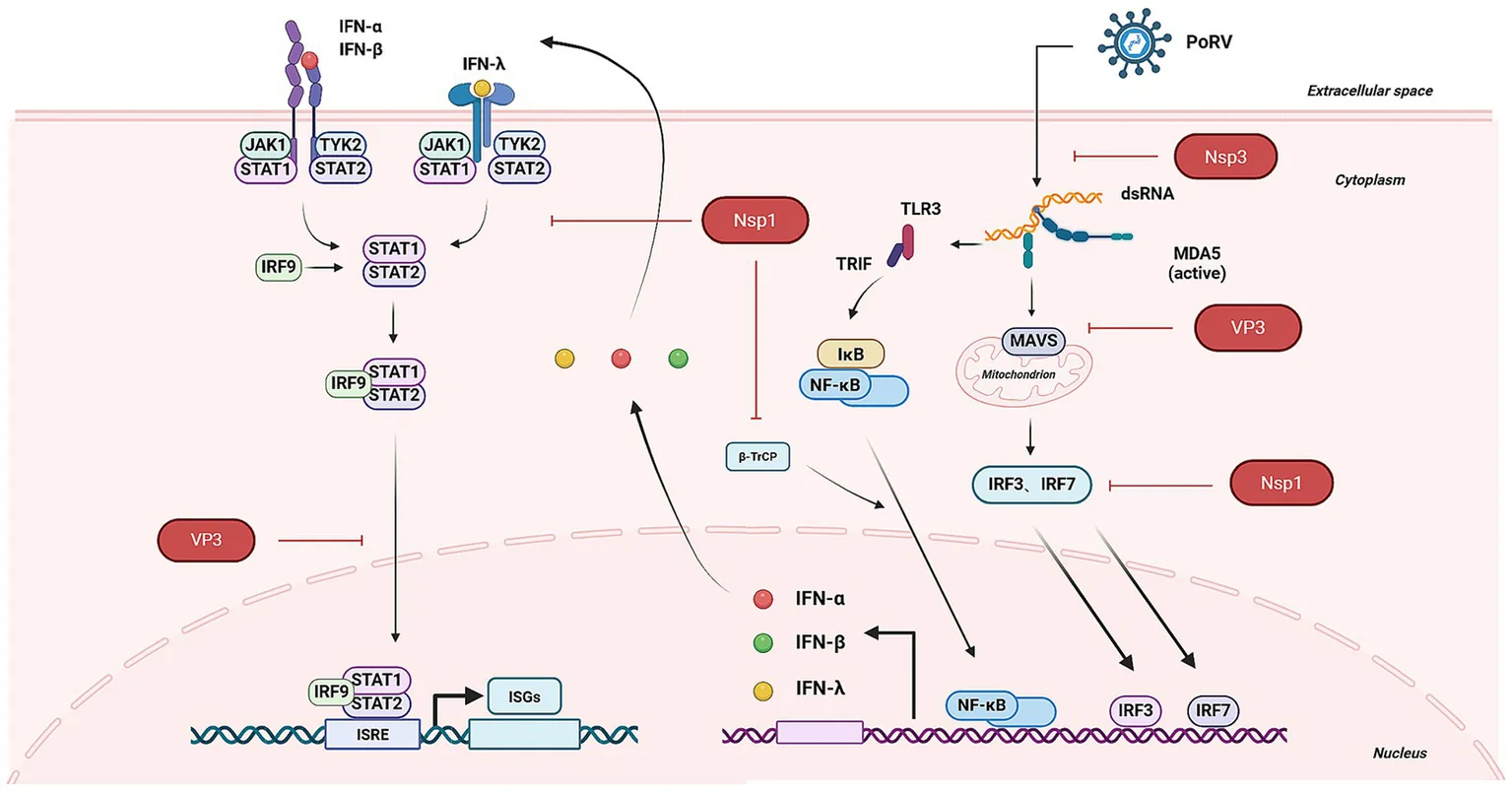
Pattern recognition receptor (PRR) signaling pathways and *PoRV* immune evasion mechanisms: During infection, host pattern recognition receptors (RIG-I/MDA-5, TLR3/7) recognize viral RNA to activate IFN production and the JAK–STAT signaling pathway, inducing antiviral states and immune regulatory responses. *PoRV* counteracts these defense mechanisms by degrading key signaling molecules through viral proteins (e.g., NSP1, VP3), thereby disrupting interferon synthesis and response pathways.

Initial immune responses are primarily executed by pattern recognition receptors (PRRs) expressed on intestinal epithelial cells (IECs) (Amarante-Mendes et al., 2018; Mogensen, 2009). IECs sense viral nucleic acids through PRRs and initiate downstream signaling cascades (Ishizuka et al., 2016) (Figure 2). Studies indicate that *PoRV* infection of porcine IECs specifically activates TLR3, RIG-I, and MDA-5, which recognize extracellular or intracellular dsRNA and transduce signals through adaptor proteins TRIF or MAVS (Pott et al., 2012; Xu et al., 2006). This pathway ultimately activates transcription factors IRF3 and NF-κB, inducing type I interferons (IFN-*α*/*β*) and interferon stimulated genes (ISGs) to establish an early antiviral state. Additionally, other PRR members including TLR5 and TLR7 participate in viral component recognition and response within the intestinal inflammatory milieu. Secreted IFN-α/β bind to their receptors in autocrine and paracrine manners, subsequently activating the JAK–STAT pathway (JAK/TYK kinases to STAT1/STAT2-IRF9 complex), which further amplifies ISG expression and directly suppresses viral replication (Li et al., 2020). IFN-*γ* activated STAT1 homodimers induce another set of ISGs, collectively constituting an antiviral network (Randall and Goodbourn, 2008; Seo and Hahm, 2010) (Figure 2). These PRR-triggered signaling cascades ultimately establish a complex antiviral network within the intestinal mucosa, composed of diverse cytokines, chemokines, and ISGs. This network not only directly curbs viral replication but also effectively recruits and activates downstream adaptive immune cells.

These signaling pathways trigger the release of a spectrum of cytokines and chemokines. *PoRV* infection induces NF-κB dependent chemokines including RANTES, IP-10, and MIP-1β (Casola et al., 1998; Rodriguez et al., 2009; Stadnyk, 2002), recruiting T cells, dendritic cells (DCs), and monocytes to infection sites (Stadnyk, 2002). Concurrently, the TLR5/NOD-like receptor C4 (NLRC4) pathway induces IL-22 and IL-18 production, enhancing epithelial barrier function and eliminating infected cells (Ouyang and Valdez, 2008; Sonnenberg et al., 2011; Xue et al., 2017; Zhang et al., 2014). DCs, as crucial antigen presenting cells, rapidly secrete type I interferons and proinflammatory cytokines upon antigen uptake, bridging innate and adaptive immunity. Other innate effector cells also contribute significantly (Holloway and Coulson, 2013; Rose et al., 1998; Youngman et al., 2002): macrophages are massively recruited to intestinal mucosa and establish early defense through IFN-α/β and TNF-α secretion (Holloway and Coulson, 2013); natural killer (NK) cells, defined in pigs as CD3^−^CD8α^+^CD2^+^ (Gerner et al., 2009), directly lyse infected cells via perforin, granzymes, and IFN-γ production [207]. Studies using B cell deficient (HCKO) pig models suggest that when CD8^+^ T cells are depleted, NK cells can compensate by enhancing IFN-γ production, particularly exerting antiviral effects locally in the intestine (Wen et al., 2016). Furthermore, porcine intestinal epithelium harbors a unique population of innate lymphoid cells (ILC-IELs) whose transcriptomic profiles correlate with cytotoxicity and tissue residency (Wiarda and Loving, 2022). These cells may be especially important during early life when adaptive immunity is immature, though their specific functions in *PoRV* infection remain to be elucidated.

### 4.2 Acquired immune response

Although innate immunity is critical in the immune response to *PoRV*, adaptive immunity ensures efficient viral clearance and provides protection against reinfection. RV infection elicits adaptive responses including antibody producing B cells and virus epitope recognizing T cells. Many antibodies against RV surface proteins VP7 and VP4 exhibit neutralizing activity *in vitro* and protective effects *in vivo*, as confirmed by passive transfer experiments in murine and gnotobiotic piglet models (Feng et al., 1997; Franco and Greenberg, 1997; Jiang et al., 2002; Offit, 1994; Yuan and Saif, 2002).

#### 4.2.1 Humoral immunity: B cells and antibody responses

**Figure 3:**
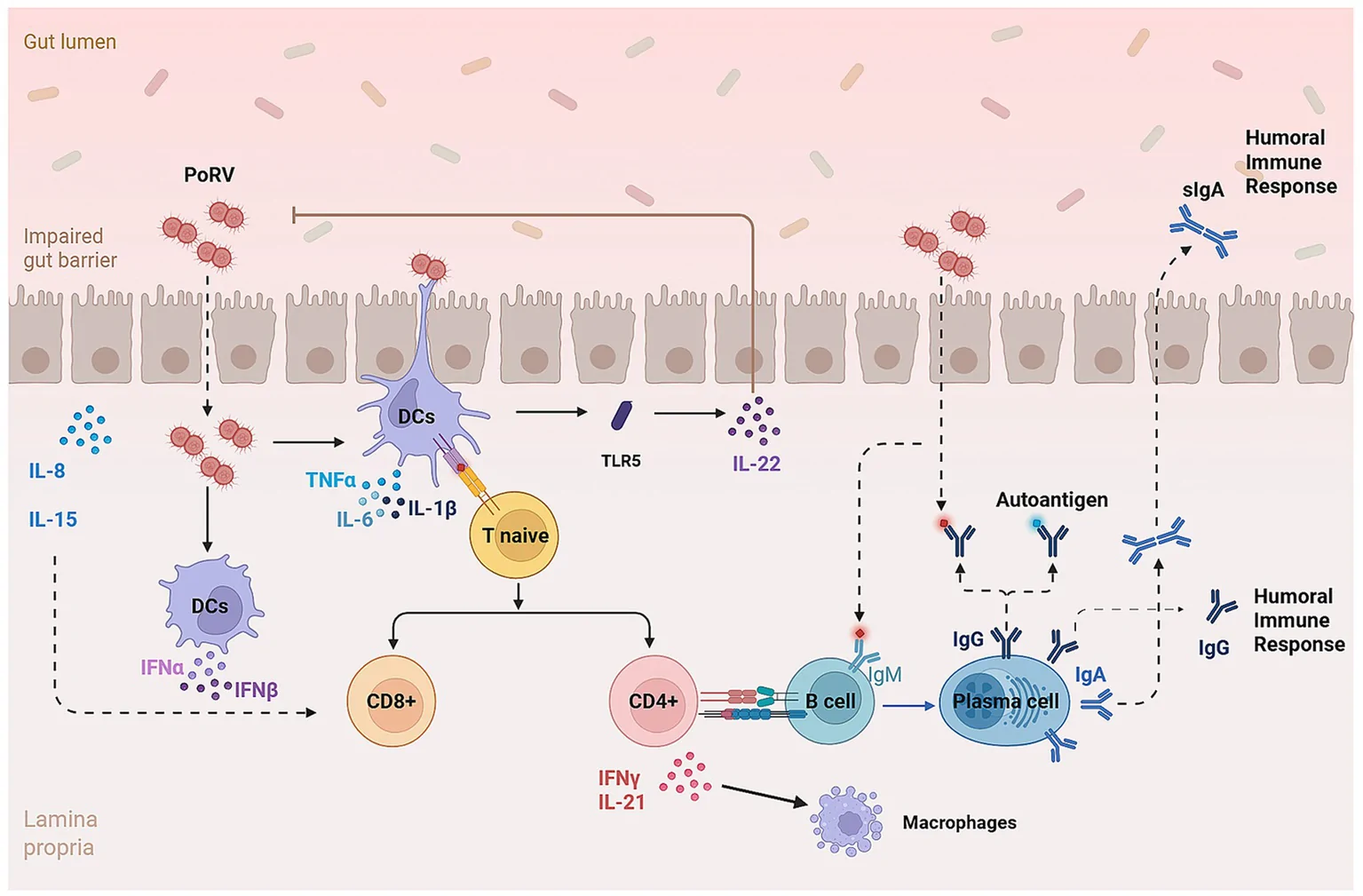
Immune response triggered by *PoRV* infection: *PoRV* invasion and replication disrupt epithelial integrity, triggering cytokine release (e.g., IL-8, IL-15) to recruit and activate dendritic cells (DCs) and macrophages. Activated antigen-presenting cells secrete proinflammatory mediators (TNF-*α*, IL-6, IL-1*β*) and type I interferons (IFN-α/β). Dendritic cells initiate primary T cell activation through multiple cytokines, driving the differentiation of CD4 + helper T cells and CD8 + cytotoxic T lymphocytes. CD4 + T cells assist B cell activation via cytokines like IFN-*γ* to produce immunoglobulins. Concurrently, protective pathways such as the TLR5/IL-22 axis are activated to enhance epithelial barrier repair.

*PoRV* infection induces systemic and local antibody responses against VP7, VP4, VP6, NSP3, and NSP4. However, only antibodies directed against VP7 and VP4 are capable of neutralizing virus (Figure 3). Passive immunity acquired through maternal colostrum and milk is crucial for protecting piglets. Colostral IgG prevents systemic infection, whereas milk secretory IgA provides local mucosal defense, a key element in *PoRV* control (Klobasa et al., 1987; Macpherson et al., 2008; Mantis et al., 2011). Field studies and research demonstrate that multiparous sows exhibit higher serum, colostral, milk, and piglet serum IgG/IgA levels compared to primiparous sows (Cabrera et al., 2012; Klobasa et al., 1986; van de Ligt et al., 2002). In pigs, IgM to IgA class switching occurs predominantly in germinal centers of gut-associated lymphoid tissue (GALT), and porcine IgA^+^ plasma cells migrate along the gut mammary axis to secrete sIgA in milk, constituting the primary route of passive protection for newborn piglets (Chabaudie et al., 1993; Saif, 1999). Maternal IgG/IgM levels gradually decline until piglets generate their own neutralizing antibodies upon active immunity establishment.

Following infection or vaccination, intestinal IgA serves as a critical correlate of protective immunity. Although serum neutralizing antibody titers are frequently measured, intestinal IgA levels more reliably predict protective efficacy (Jaimes et al., 2002). Studies using B cell deficient (HCKO) pig models further confirmed the essential role of B cells in vaccine induced protective immunity: compared to wild type pigs, HCKO pigs exhibited significantly prolonged viral shedding duration and higher diarrhea incidence upon *Human Rotavirus* (*HRV*) challenge, indicating that B cells and their derived antibodies are indispensable for controlling infection and preventing disease (Wen et al., 2016). Cross protection against different *PoRV* strains may exist. While homologous immunity (against the infecting strain) is most potent, heterologous protection is mediated by antibodies targeting cross reactive epitopes on VP4 (especially the VP5* region) and VP6, and does not entirely depend on serum neutralizing antibody titers (Angel et al., 2012; Clarke and Desselberger, 2015; De Vos et al., 2009; Hoshino et al., 2004; Nair et al., 2017; Ruiz-Palacios et al., 2006; Shao et al., 2016).

#### 4.2.2 Cellular immunity: T-cell responses

Multiple immune cells mediate adaptive immunity against *PoRV*. Following antigen presentation by dendritic cells (DCs), CD4^+^ Th cells secrete IFN-*γ* to directly suppress viral replication and assist B cell activation to promote intestinal IgA production (Vlasova et al., 2017). A gnotobiotic pig study demonstrated that the frequency of intestinal IFN-γ producing T cells (CD4^+^/CD8^+^) correlated significantly positively with protection against RV diarrhea (*r* = 0.97–1.0, *p* < 0.005) (Yuan et al., 2008). Furthermore, depletion studies revealed compensatory mechanisms within the immune network: when CD8^+^ T cells were depleted, CD4^+^ T cells provided alternative protection through IFN-γ/IL-21 mediated macrophage activation (Jaimes et al., 2002; Wu et al., 2011).

CD8^+^ cytotoxic T lymphocytes (CTLs) eliminate infected intestinal epithelial cells through their cytotoxic activity, thereby limiting viral spread across mucosal surfaces (Vlasova et al., 2017). Data from specific gene knockout (HCKO) pig models prove that CD8^+^ T cell depletion leads to elevated peak viral shedding (from 43 to 944 FFU/mL), prolonged diarrhea duration (from 1.3 to 3.7 days), and markedly delayed viral clearance (Wen et al., 2016). A 2024 study in weaned piglets further showed that nutritional intervention through supplemental spray dried plasma increased intestinal intraepithelial CD8^+^ T cell numbers, significantly enhancing mucosal immunity and promoting viral clearance, providing additional evidence for the protective role of CD8^+^ CTLs in porcine intestinal mucosal immunity.

A notable limitation is that data directly addressing *PoRV*-specific immune responses remain scarce. Current understanding of RV immune mechanisms is largely derived from studies using gnotobiotic piglets as models for *HRV* infection and immune responses. While this model is valuable, the unique strain diversity and host adaptation of *PoRV* strongly suggest that the immune response characteristics it elicits may differ from those of *HRV*. Therefore, there is an urgent need for future studies to systematically generate *PoRV*-specific immunological databases that comprehensively characterize antibody and T-cell response dynamics, establish correlates of protection for *PoRV*, and assess the true extent of cross-protection among different *PoRV* strains (G and P types). Such information is critical for the development of effective *PoRV* vaccines.

### 4.3 Immune escape mechanism

**Figure 4:**
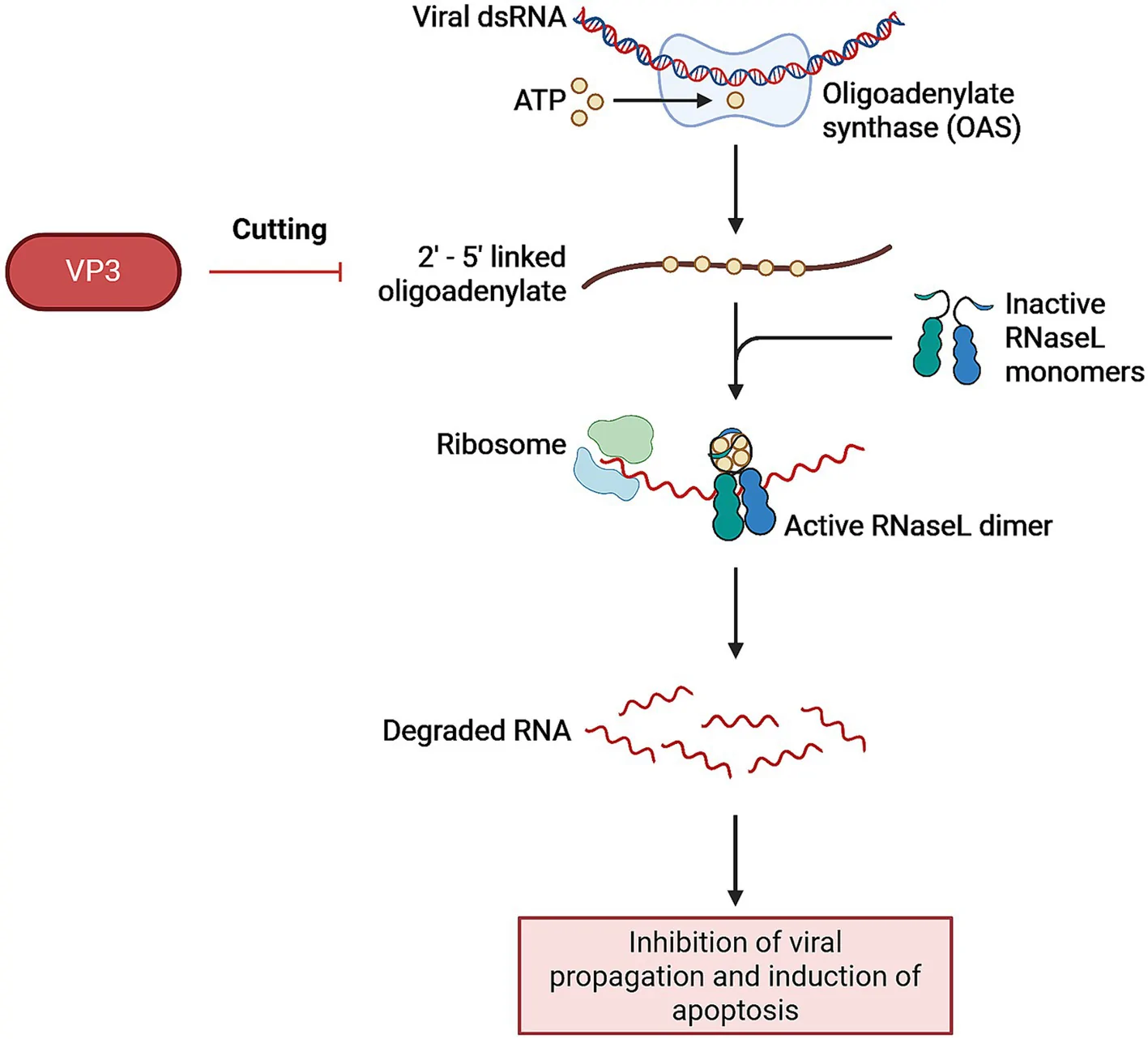
*PoRV* VP3 protein disrupts the RNase L antiviral pathway.

Despite the host mounting complex antiviral immune responses, *PoRV* has evolved multiple immune evasion strategies to counteract host defenses, thereby enabling persistent infection. (1) NSP3 achieves host translational shutdown by inhibiting cellular mRNA translation: Poly(A)-binding proteins (PABPs) are an important family of gene expression factors that are common targets of viruses. PABPs function as both translation factors and key mRNA metabolism regulators. Viruses manipulate PABP activity to subvert translation or evade antiviral defenses (Gao et al., 2022). While eIF4G mediates poly(A)^+^ mRNA capping/cyclization and the PABP-eIF4G complex initiates translation, NSP3 competes with PABP for eIF4G binding. Poncet et al. demonstrated NSP3 binds viral mRNAs via 3’ GACC motifs, competing with host translation initiation (Gratia et al., 2016). (2) Suppression of IFN production: The viral protein NSP1 blocks IFN transcription by degrading key transcription factors such as IRF3, IRF5, IRF7, and IRF1 (Arnold and Patton, 2011; Feng et al., 2009); VP3 suppresses ISG expression by degrading MAVS to inhibit the RIG-I/MDA-5 pathway (Ding et al., 2018) or by competitively disrupting the PFDN-UBA3-IRF9 axis (Fan et al., 2002; Geissler et al., 1998; Wong et al., 2023; Zhao et al., 2021; Zhu et al., 2025). (3) Interfering with IFN downstream signaling: NSP1 induces *β*-TrCP degradation, destabilizing IκB and thereby suppressing NF-κB-dependent IFN responses. Simultaneously, it impedes STAT1/STAT2 activation, rendering cells “desensitized” to IFN stimulation (Di Fiore et al., 2015; Graff et al., 2009; Lutz et al., 2016) (Figure 2). (4) Antagonizing antiviral pathways: VP3 can cleave 2′,5′-oligoadenylate (2-5A) (an activator of the RNase L pathway), evading this antiviral mechanism (Morelli et al., 2015) (Figure 4). NSP4 binds LC3 to inhibit autophagy, evading this innate clearance mechanism (Berkova et al., 2006). Additionally, the virus modifies its own RNA by hijacking the host’s m^6^A methylation system to mimic host RNA, thereby evading detection by pattern recognition receptors (Bian et al., 2020; Dominissini et al., 2012; Hao et al., 2019; Lichinchi et al., 2016; Liu et al., 2014; Lukhele et al., 2022; Schoggins et al., 2011).

## 5 The zoonotic potential and cross-species transmission of PoRV

The potential for zoonotic transmission of *PoRV* is increasingly evident, primarily achieved through genetic recombination and direct cross-species transmission, posing a potential threat to the protective efficacy of existing vaccines.

### 5.1 Genotype distribution and evidence of recombination genetics

Porcine and human *Rotavirus* A (RVA) strains differ in their common genotype profiles. Common genotypes in porcine isolates include G3, G4, G5, G9 and P[6], P[7], P[13], P[19]. A recent review compiled data identifying ten G genotypes (G1-G5, G9-G12, and G26) and seven P genotypes (P[4], P[6], P[8], P[13], P[14], P[19], and P[25]) as definitively of porcine origin (Ghonaim et al., 2024; Vlasova et al., 2017). In contrast, human RVA strains are predominantly characterized by G1, G2, G3, G9 and P[8], P[4]. Numerous porcine-origin RVA strains have been detected in human patients, indicating that swine-derived human RVA strains have achieved more successful emergence and transmission within human populations than reassortants from other animal reservoirs (Martella et al., 2010; Vlasova et al., 2017; Wu et al., 2022).

Reassortment events continuously blur interspecies barriers at the molecular level (Fujiwara and Nakagomi, 1997; Iizuka et al., 1994). For example, the P[19] genotype was initially identified in a porcine G3P[19] strain in China and subsequently detected in human samples (G9P[19]) from Thailand, India, and Vietnam (Burke et al., 1994; Saikruang et al., 2013). Moreover, G5, the most common genotype in swine, has been frequently detected in children in several South American countries (Da Silva et al., 2011), providing early serological and epidemiological clues for direct cross-species transmission of *PoRV*. Phylogenetic analysis further revealed high homology between porcine and human VP7 gene sequences of G3, G4, G5, G9 and sequences of P[6], P[13], P[19] (Ndebe et al., 2023; Wu et al., 2022).

Whole-genome studies have revealed more complex cross-species gene flow. Recently, Chinese researchers isolated two G9P[23] strains with the whole-genome constellation G9-P[23]-I5-R1-C1-M1-A8-N1-T7-E1-H1, which overall exhibited porcine lineage characteristics. However, phylogenetic analysis showed that their VP7 genes clustered closely with human G9 strains, suggesting that these gene segments may have undergone reassortment with human strains (Ghonaim et al., 2024). The G11P[25] strain detected in Korea (genotype G11-P[25]-I12-R1-C1-M1-A1-N1-T1-E1-H1) was identified as a reassortant of two distantly related swine-human strains of the G11 lineage (Than et al., 2013). The G12P[7] (CN127) and G9P[19] (GDJM1) strains from China, as well as the G9P[13] and G4P[6] strains from Mozambique, have been confirmed to result from multiple reassortments between porcine and human viral gene segments (Boene et al., 2021; Luo et al., 2023; Miao et al., 2022). Molecular epidemiological studies have provided quantitative evidence for this homology. Studies from India showed that the VP7 genes of porcine G4 and G9 strains shared nucleotide similarities of 96.83 and 98.89% with human strains, respectively, while the NSP3 (T1) and NSP4 (E1) genes also exhibited human-associated characteristics (Arias et al., 2002). Similarly, the G9P[19] strain GDJM1 from Guangdong, China, showed high homology (91.83–96.69%) with human strains in seven gene segments (Luo et al., 2023). A study in Taiwan (2014–2017, 153 farms) indicated that the NSP4 (E1) gene shared 88.4% homology with the human G4P[6] strain 03–98 s-140 isolated in 2009 (Wu et al., 2022).

Notably, cross-species reassortment and gene homology are not confined to the porcine-human interface. Whole-genome analysis of the *Bovine Rotavirus (BRV)* isolate 0205HG (G6P[1]) from China revealed features of multiple-source reassortment across its 11 gene segments. Most genes showed high homology with bovine strains, VP1, VP4 and NSP5 were most closely related to *HRV*, and VP3 exhibited high identity (90.51%) with a *Feline Rotavirus (FRV)*. This finding suggests that *BRV* is also involved in the exchange of gene segments with human and *FRV* under natural conditions (Ghonaim et al., 2025a, 2025b). In addition, studies on *Equine Rotavirus (ERV)* have demonstrated that equine-like G3 rotaviruses have been detected in paediatric cases in multiple countries, and *ERV* genomes have also been found to contain gene segments from other hosts such as bovine and feline sources. Together, these observations indicate that cross-species reassortment events occur extensively among major livestock species including swine, cattle and horses, as well as companion animals like cats, and humans, rather than being restricted to a single animal species (Ghonaim et al., 2025a, 2025b).

### 5.2 Global spread trends and dynamics of dominant genotypes

Beyond the context of genetic reassortment, the global transmission dynamics of specific genotypes further increase the complexity of cross-species transmission. Recent global epidemiologic studies confirm *PoRV* G9 genotype dominance, which demonstrates rapid global dissemination potential and high VP7 homology with human G9 strains (Matthijnssens et al., 2010; Teodoroff et al., 2005). An analysis of US *PoRV* strains from 2004 to 2012 revealed that G9P[13] became the most prevalent genotype, accounting for 60.9%, while the historically most common G5 type no longer dominated (Vlasova et al., 2017). In Korea (2014–2018), G4 and G9 were the most common G types (each accounting for 39.6%), while P[7] and P[6] were the most common P types (Truong et al., 2022). In China (2017–2024), prevalent strains included G9P[7], G5P[13], G9P[13], G5P[7], G9P[23] and G9P[6]. G9P[13] was also widely detected in surveys conducted in India and Mozambique (Boene et al., 2021). Some genotypes, represented by G9, not only possess ecological advantages for rapid transmission, but also frequently carry gene segments in their genetic backbones that are highly similar to human strains. Meanwhile, the predominance of G5 in the United States has been replaced, and strains such as G1P[7] and G12P[7] have emerged in China (Kim et al., 2012; Miao et al., 2022; Okitsu et al., 2011; Wu et al., 2017). These dynamic changes collectively illustrate that the predominant genotypes of *PoRV* are undergoing complex spatiotemporal evolution, further enriching their genotype diversity and adding new targets for future surveillance.

### 5.3 Public health implications and the future of “one health” surveillance

The above genetic evidence suggests a potential risk of cross-species transmission of *PoRV*, and direct zoonotic transmission of *PoRV* has been confirmed in multiple countries (Holmes et al., 1999). Porcine-originated RVA strains were identified in children with acute gastroenteritis (AGE) in Kenya, DRC, Egypt, Thailand, China, and Argentina (Degiuseppe et al., 2013; Zhou et al., 2015). Limited evidence indicates that porcine RVB may also possess zoonotic transmission (Medici et al., 2010). However, no evidence of swine-to-human transmission has been identified for RVC/RVH to date. Combining genetic and epidemiological clues, the most explicit public health concern currently remains focused on porcine RVA. This situation highlights the necessity of strengthening systematic surveillance of rotaviruses in swine herds. In-depth investigation of their genetic diversity will not only help assess the evolutionary trends of cross-species transmission, but also provide a scientific basis for early warning of emerging strains and optimization of vaccination strategies. Meanwhile, continued attention to reassortment events in rotaviruses from other livestock such as cattle and horses, as well as companion animals, should be an integral component of multi-host joint surveillance under the One Health framework. When evaluating such risks, it is necessary to strictly distinguish documented clinical zoonotic transmission events from potential risks suggested by genetic evidence, in order to avoid overinterpretation of current public health risks. Establishing an integrated multi-host surveillance system will help to more precisely differentiate between genetic noise and real threats, thereby providing objective scientific guidance for vaccine strain selection and public health policy formulation.

## 6 Prevention and control

*RoRV* infections are widespread in swine herds and difficult to eradicate. Therefore, the core of prevention and control strategies lies in reducing morbidity and mortality through comprehensive measures, among which maintaining and enhancing sow immunity is critical. Effective biosecurity measures and all-in-all-out management system serve as the foundation for preventing the invasion and spread of *PoRV*. On this basis, the current core immunization strategy is to enhance sow immune status through vaccination, thereby providing high levels of colostral antibodies (especially sIgA) to piglets for passive protection against early infection.

However, the efficacy of current vaccination strategies is facing multiple serious challenges. Currently, the United States Department of Agriculture (USDA) has approved two vaccines for preventing RVA in swine: ProSystem Rota and ProSystem RCE (Kumar et al., 2022). China mainly uses a trivalent live vaccine against *TGEV*, *PEDV* and *PoRV*, which is based on a G5 strain. Although these vaccines have been applied in production, field efficacy data are limited, making it difficult to accurately estimate their effectiveness. More critically, circulating genotypes continue to drift. For example, in the United States and China, G9 has replaced the classic G5 as the predominant strain. The antigenic mismatch between traditional vaccines based on old strains and field epidemic strains has directly led to a marked decline in protective efficacy. In addition, conventional live attenuated vaccines carry potential risks of reversion to virulence and reassortment with wild-type strains, which may paradoxically increase viral genetic diversity (Ghonaim et al., 2025a, 2025b; Li et al., 2024). Another notable issue is that maternal antibody interference poses a major obstacle to active immunization. The high titers of maternal antibodies commonly present in newborn piglets can neutralize oral live vaccine viruses during active immunization, limiting their effective replication in the intestine and thus severely impairing the establishment of early active immunity. These challenges are driving researchers to explore new, safer, and more effective prevention and control strategies, with the research focus shifting from traditional live attenuated vaccines to multivalent designs, novel delivery platforms, and non-replicating antigen vaccines (Ghonaim et al., 2025a, 2025b).

In terms of optimizing conventional live attenuated vaccines, scientists have developed an oral trivalent live attenuated vaccine using the Korean predominant strains G8P[7], G9P[23] and G5P[7], and verified that it can safely induce specific antibodies in piglets and effectively alleviate diarrhea symptoms (Park et al., 2019), providing a successful example for timely updating of vaccine strains. Meanwhile, based on the understanding of *RV* entry mechanisms, the structural proteins VP4 and VP7 and their trypsin-cleaved fragment VP8, which play key roles in viral entry and neutralizing antibody responses, have become core antigen targets for novel subunit vaccine development (Ghosh et al., 2012). Consequently, multiple technical approaches have been derived. The recombinant plasmid pPI-2. EGFP. VP7. S, constructed based on the *PoRV* VP7 gene and the PEDV S gene, demonstrated good immunogenicity in a mouse model, inducing both specific humoral and cellular immune responses against both viruses simultaneously. Multivalent mucosal vaccines using live vectors such as recombinant lactic acid bacteria can simultaneously express antigens of multiple pathogens, including *PEDV*, *TGEV*, and *PoRV* (Guo et al., 2022), providing a novel strategy for controlling mixed infections (Hou et al., 2018; Zhou et al., 2019).

Beyond subunit vaccines, virus-like particle (VLP) vaccines have attracted considerable attention due to their spatial conformation similar to native viruses and their excellent safety profile (Jennings and Bachmann, 2009). By co-expressing VP2, VP6 and VP7 using the baculovirus-insect cell expression system, these proteins can self-assemble into VLPs with morphology highly similar to native viruses. Animal studies have confirmed that these VLPs can induce not only potent neutralizing antibodies but also cellular immunity represented by IFN-*γ* secretion (Laimbacher et al., 2012). More importantly, because VLP vaccines do not contain the complete viral genome and do not replicate in the host, they can theoretically effectively bypass maternal antibody interference, which gives them a unique advantage in early immunization of newborn piglets. Messenger RNA (mRNA) vaccines, characterized by short development cycles, convenient industrial production, and the ability to activate both humoral and cellular immunity, have emerged as promising third-generation nucleic acid vaccines (Lu et al., 2024). VP7-mRNA vaccines have been shown to efficiently activate antigen-specific CD8^+^ T cells and promote IFN-γ secretion, thereby mediating robust antiviral cellular immunity (Lu et al., 2024), and they also possess theoretical advantages in bypassing maternal antibody interference (Ghonaim et al., 2025a, 2025b). In addressing the challenge of specific RVC is the most common cause of *RV* diarrhea in piglets under 1 week of age. However, because RVC is difficult to adapt to *in vitro* cell culture, no commercial conventional vaccine against RVC is available to date. To overcome this obstacle, a viral-vectored platform technology called Sequivity has been developed, which allows rapid construction of customized vaccines directly using the VP7 sequences of circulating field strains, thereby bypassing the bottleneck of virus isolation and culture in traditional vaccine development. Early field trials indicated that, compared with natural exposure, this approach significantly reduced piglet mortality and increased daily weight gain. This concept validates the potential of sequence-based rapid vaccine design in the veterinary field. However, its exact efficacy in preventing RVC in swine herds still requires validation through larger-scale studies. Concurrently, immunoinformatics and molecular dynamics simulation technologies can be employed to design broad-spectrum multivalent vaccines targeting conserved epitopes on proteins such as VP7 and VP8 (Albaqami et al., 2023; Joshi et al., 2023; Paul et al., 2023; Zhu et al., 2024). Simulations have verified their theoretical stability upon binding to immune receptors, laying a critical foundation for the development of next-generation precise and efficient vaccines (Li et al., 2014). It should be emphasized that although the conformational stability and affinity of these computer-predicted candidate epitopes upon binding to immune receptors have received preliminary validation through molecular dynamics simulations, whether they truly possess immunogenicity and protective efficacy still requires further verification through *in vivo* experiments in animal models such as mice and piglets. This translational gap from computational prediction to biological validation represents a critical bottleneck that current epitope vaccine development must overcome.

Overall, the advanced technologies described above represent key future trends in *PoRV* vaccines (Yan et al., 2024). Despite substantial progress in the research and implementation of *PoRV* vaccines, several critical challenges remain to be addressed to further enhance their global impact. Broader genotype coverage is needed, as circulating strains continue to evolve; vaccine strains should be regularly updated or adopt multivalent and cross-protective antigen designs. Safer, non-replicating vaccines should be developed to avoid the risks of live vaccine reversion to virulence and recombination, with novel subunit, VLP and mRNA vaccines representing important directions. Field surveillance and efficacy evaluation should be strengthened to clarify the actual protective efficacy of existing vaccines in commercial swine herds. The introduction of reverse genetics systems would facilitate accelerated construction and evaluation of novel vaccine strains. Vaccination strategies for *PoRV* need to be established. Although attention to *PoRV* vaccines is increasing, substantial differences exist in specific vaccine strains, immunization schedules and implementation programs; establishing more standardized approaches could optimize the effectiveness of these vaccines. Finally, continued research into next-generation *PoRV* vaccine technologies, such as mucosal or needle-free formulations, holds promise for improving administration convenience, immunogenicity and overall efficacy.

**Figure 5:**
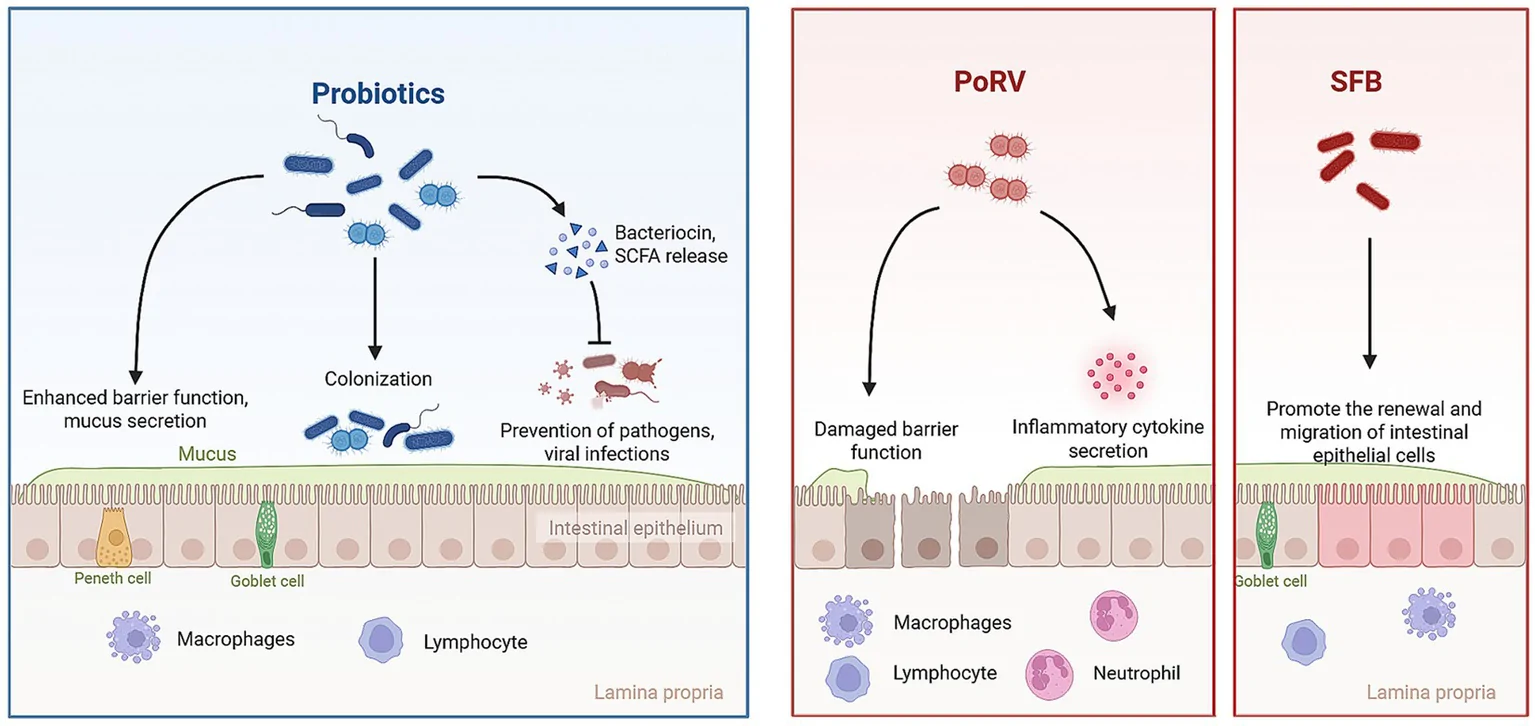
Comparison of gut microbiota-mediated defense mechanisms against *PoRV* infection: *PoRV* infection disrupts epithelial barrier function and induces inflammatory cytokine secretion. Probiotics enhance barrier integrity through competitive exclusion and immunomodulation, thereby limiting *PoRV* invasion and pathogenic effects. SFB exerts protective effects via non-immune mechanisms by directly promoting epithelial cell renewal and migration, accelerating the physical shedding of susceptible intestinal cells.

In addition to the iterative upgrading of vaccine technologies described above, interventions targeting the gut microbiota and natural bioactive substances have become important supplementary control measures. Probiotics (e.g., LGG and *L. acidophilus*) can shorten the duration of *RV* diarrhea and reduce viral shedding through competitive inhibition, strengthening the intestinal barrier, and immune modulation, while also enhancing the immunogenicity of oral vaccines (Borchers et al., 2009; Guandalini, 2006, 2008; Guandalini et al., 2000; Guarino et al., 1997; Isolauri et al., 1991; Sanders and Klaenhammer, 2001; Schusterova et al., 2024; Teran et al., 2009). Recent studies indicate that segmented filamentous bacteria (SFB) promote the renewal and migration of intestinal epithelial cells, accelerating the shedding of susceptible cells and thereby preventing infection through physical barrier mechanisms independent of adaptive immunity (Shi et al., 2019) (Figure 5). Bacterial metabolites such as neuraminidase can interfere with viral receptors (Diaz-Salinas et al., 2013), while the plant-derived purslane polysaccharide (POL-P) inhibits key proteins involved in viral entry and replication by regulating the levels of interferons and inflammatory cytokines (Zhou et al., 2023). Although these mechanistic studies are encouraging, field application evidence is currently insufficient. Relative to core vaccination strategies, these approaches are more suitable as prospective research directions rather than primary clinical intervention tools at present. In the future, if these microbiome-modulating and natural product interventions can be rationally integrated with novel vaccine strategies, a more comprehensive multi-layered prevention and control system may be established.

Overall, from epidemic strain updating, multivalent vector delivery, VLP/mRNA self-assembly and RNA particle platforms, to immunoinformatics-driven epitope design and microbiome-assisted interventions, the synergistic advancement of these cutting-edge technologies is laying a solid foundation for developing next-generation *PoRV* control systems that are more efficacious, safer, and capable of addressing complex field epidemics.

## 7 Discussion

*PoRV* is an important pathogen causing piglet diarrhea, and its complex pathogenic mechanisms and host immune interaction networks remain core research issues. Although significant progress in *PoRV* research has been made in recent years, driven by advances in molecular biology and immunological techniques, key issues such as the high degree of viral variability, the spatiotemporal specificity of the host immune response, and the susceptibility of young animals remain incompletely elucidated. This review systematically summarizes current knowledge on *PoRV* epidemiology, pathogenic mechanisms, host immune responses, immune evasion, cross-species transmission, and control strategies, and proceeds to propose several critical scientific questions and control insights.

The sophisticated multi-receptor relay entry strategy of *PoRV*, which underlies its tissue tropism and pathogenesis, not only explains the high susceptibility of neonatal piglets but also identifies critical vulnerable targets for developing post-attachment neutralization strategies. The discovery that certain bacterial neuraminidases can inhibit infection by cleaving SA receptors presents a novel, microbiota-based antiviral concept. However, the precise stoichiometry and spatiotemporal regulation of these receptor interactions in the complex gut milieu *in vivo* remain poorly defined. Future work employing intestinal organoids and single-cell transcriptomics of infected tissue will be crucial to map these dynamics and assess their variation across dominant field strains (e.g., G9 vs. G5), which is essential for designing broad-spectrum entry inhibitors.

Following successful entry, *PoRV* employs a dual strategy of direct cytolysis and enterotoxin (NSP4)-mediated secretory dysfunction to induce diarrhea. The observed viremia and detection of viral RNA in extra-intestinal sites (e.g., lung, liver) suggest systemic dissemination, a phenomenon with significant implications for disease severity and potential extra-intestinal complications. While replication in these sites appears limited, the inflammatory sequelae could contribute to overall morbidity. The host compensatory response, characterized by crypt hyperplasia and increased protein synthesis, underscores the intestine’s remarkable regenerative capacity but may also temporarily disrupt its absorptive function. A key unresolved question is how viral factors like the m^6^A methylation hijacking by VP6 precisely modulate host cell translation and innate immune gene expression to favor replication. Integrating epigenomic analyses with metabolomic studies of infected enterocytes will illuminate these immune-evasion tactics and identify new therapeutic nodes.

The host immune response to *PoRV* is a tightly coordinated but age-compromised process. In neonates, the rapid waning of maternal systemic IgG, coupled with its inefficiency in the gut lumen, creates a perilous susceptibility window. Our synthesis confirms that durable protection correlates strongly with mucosal IgA, which is best stimulated by local infection or effective mucosal vaccines. The demonstrated adjuvant effect of NSP4 in enhancing VP4/VP6-specific mucosal immunity offers a promising strategy for next-generation vaccine design. Furthermore, the compensatory role of T-cell immunity, particularly IFN-*γ*-secreting CD4^+^ and CD8^+^ T cells in viral clearance—evident in B-cell-deficient models—highlights the need for vaccines that robustly engage cellular arms. Current vaccine limitations stem primarily from genetic drift and the poor cross-neutralization between serotypes. Therefore, platforms capable of presenting conserved epitopes from multiple proteins (VP4, VP6, VP7), such as the promising VLP and computationally designed multi-epitope vaccines, represent a logical and necessary evolution. More importantly, the true level of cross-protection among different G/P-type *PoRV* strains, as well as the relative contribution of T cell immunity—particularly tissue-resident memory T cells—to protection, have so far lacked *PoRV*-specific data. Addressing this question bears directly on the selection of correlates of vaccine efficacy and on the immunogenicity design criteria for next generation broad spectrum vaccines.

The zoonotic potential of *PoRV*, particularly RVA, constitutes a significant One Health concern. The phylogenetic intermingling of porcine and human strains, exemplified by G9P[19] and G4P[6] recombinants, provides concrete evidence of cross-species transmission. However, the actual public health burden of such transmission has not been systematically assessed, and the molecular restrictions governing cross-species transmission—including receptor specificity, replication fitness, and interspecies differences in host antiviral factors—remain largely undefined. Under the current One Health framework, this represents a scientifically urgent question with practical implications. Such genetic reassortment not only threatens public health but also directly compromises vaccine efficacy in both species by introducing novel strains capable of evading existing immunity. Consequently, prevention strategies must extend beyond farm-level biosecurity—which remains foundational—to include integrated surveillance. Genomic monitoring of circulating *PoRV* strains in swine must be routine to promptly identify emerging zoonotic variants. The economic imperative is clear: beyond direct mortality, *PoRV* inflicts substantial costs through growth retardation and treatment, demanding more effective interventions. The integration of targeted probiotic supplementation (e.g., LGG) and vaccine responses, alongside advanced vaccines, forms a holistic mitigation framework.

Breakthroughs in the aforementioned unresolved questions hinge largely on the introduction and application of emerging research technology platforms. Traditional *PoRV* research has relied primarily on immortalized cell lines or live animal models—the former fail to recapitulate the cellular heterogeneity and three-dimensional architecture of the intestinal epithelium, whereas the latter suffer from high cost, low throughput, and ethical constraints. The advent of porcine intestinal organoid technology offers a transformative solution to this predicament: these organoids retain the cellular diversity, regional specificity (duodenum vs. ileum vs. colon), and genetic background of donor pigs, providing a near-physiological *in vitro* platform for investigating *PoRV* cell tropism, receptor utilization, replication kinetics, and innate immune responses of the intestinal epithelium. More importantly, organoids can be derived from pigs of different ages, thus enabling direct dissection of the cell-autonomous mechanisms underlying age-dependent susceptibility. Concurrently, advances in single-cell and spatial transcriptomics allow researchers to deconstruct, at the individual cell level, the response profiles of intestinal epithelial stem cells, absorptive enterocytes, goblet cells, Paneth cells, enteroendocrine cells, and various immune cell types within infected tissues, and to map gene expression data back to the tissue architecture *in situ*, revealing the spatiotemporal dynamics of intercellular communication within the infection microenvironment. These technologies hold substantial promise for elucidating the mechanisms of intestinal epithelial repair and the regulation of local immune networks following *PoRV* infection. At the structural biology front, continued progress in cryo-electron microscopy (cryo-EM) and X-ray crystallography has rendered it feasible to resolve VP4/VP7 conformational changes, the pore-forming mechanism of NSP4, and the fine details of antibody recognition of neutralizing epitopes—offering the prospect of atomic-level guidance for antiviral drug design and broad spectrum vaccine antigen optimization.

In conclusion, *PoRV* is a pathogen of both fundamental research value and urgent practical importance for disease control. The sophisticated “attack-defense-countermeasure” interplay between the virus and its host provides an ideal model for elucidating the basic principles of virus-host interactions, whereas its remarkable genetic diversity and cross-species transmission potential pose a sustained and ever-evolving threat to livestock production and public health. Controlling *PoRV* requires a dual-front approach: deepening fundamental research into virus-host dynamics to identify novel antivirals and immune correlates of protection, and translating these insights into applied tools like broadly protective multivalent vaccines and robust management protocols. Bridging the gap between veterinary and H*RV* research will be mutually beneficial, accelerating the development of superior mucosal vaccines and adjuvants. Future efforts must prioritize interdisciplinary studies that combine genomics, immunology, and herd health management to mitigate the economic and zoonotic threats posed by this ever-evolving pathogen.

## Glossary

PoRV: *Porcine Rotavirus*
RV: Rotavirus
RVA–RVH: Rotavirus species A–H
HRV: Human Rotavirus
BRV: Bovine Rotavirus
ERV: Equine Rotavirus
FRV: Feline Rotavirus
TLP: Triple-Layered Particle
DLP: Double-Layered Particle
SA: Sialic Acid
HBGA: Histo-Blood Group Antigen
Hsc70: Heat Shock Cognate Protein 70
SOCE: Store-Operated Calcium Entry
ER: Endoplasmic Reticulum
PRR: Pattern Recognition Receptor
TLR3/5/7: Toll-Like Receptor 3/5/7
RIG-I: Retinoic Acid-Inducible Gene I
MDA-5: Melanoma Differentiation-Associated Protein 5
MAVS: Mitochondrial Antiviral Signaling Protein
TRIF: TIR-Domain-Containing Adapter-Inducing Interferon-β
IRF3/7: Interferon Regulatory Factor 3/7
NF-κB: Nuclear Factor Kappa-B
IFN: Interferon
ISG: Interferon-Stimulated Gene
JAK–STAT: Janus Kinase–Signal Transducer and Activator of Transcription
IEC: Intestinal Epithelial Cell
DC: Dendritic Cell
NK Cell: Natural Killer Cell
ILC-IEL: Innate Lymphoid Cell–Intraepithelial Lymphocyte
CTL: Cytotoxic T Lymphocyte
GALT: Gut-Associated Lymphoid Tissue
sIgA: Secretory Immunoglobulin A
PABP: Poly(A)-Binding Protein
eIF4G: Eukaryotic Initiation Factor 4G
β-TrCP: Beta-Transducin Repeat-Containing Protein
2-5A: 2′,5′-Oligoadenylate
RNase L: Ribonuclease L
LC3: Microtubule-Associated Protein 1A/1B-Light Chain 3
m^6^A: N^6^-Methyladenosine
VLP: Virus-Like Particle
mRNA: Messenger RNA
PEDV: Porcine Epidemic Diarrhea Virus
TGEV: Transmissible Gastroenteritis Virus
SFB: Segmented Filamentous Bacteria
LGG: Lactobacillus rhamnosus GG
POL-P: Purslane Polysaccharide
USDA: United States Department of Agriculture
AGE: Acute Gastroenteritis
HCKO: Homozygous disruption in the gene encoding immunoglobulin heavy chain
SPF: Specific Pathogen-Free
Cryo-EM: Cryo-electron microscopy
